# Association of Basal Serum Androgen Concentration with Follicles Number on the Day of Triggering Final Oocyte Maturation in Low Responders According to the Bologna Criteria: A Prospective Cohort Study

**DOI:** 10.3390/ijms26041656

**Published:** 2025-02-15

**Authors:** Julia K. Bosdou, Christos A. Venetis, Panagiotis Anagnostis, Despoina Savvaidou, Katerina Chatzimeletiou, Leonidas Zepiridis, Dimitrios G. Goulis, Grigoris Grimbizis, Efstratios M. Kolibianakis

**Affiliations:** 1 Unit for Human Reproduction, 1st Department of Obstetrics and Gynecology, Medical School, Aristotle University of Thessaloniki, 56429 Thessaloniki, Greece; juliabosdou@gmail.com (J.K.B.); venetis@gmail.com (C.A.V.); sabbaidu@gmail.com (D.S.); katerinachatzime@hotmail.com (K.C.); leozepi4@gmail.com (L.Z.); grigoris.grimbizis@gmail.com (G.G.); stratis.kolibianakis@gmail.com (E.M.K.); 2 Unit of Reproductive Endocrinology, 1st Department of Obstetrics and Gynecology, Medical School, Aristotle University of Thessaloniki, 56429 Thessaloniki, Greece; dimitrios.goulis@gmail.com

**Keywords:** androgens, testosterone, DHEAS, low responders, follicles

## Abstract

Studies in animals have shown that androgens promote early follicular development and granulosa cell proliferation by augmenting follicle-stimulating hormone (FSH) receptor expression in granulosa cells. Inconsistency exists regarding the association between basal serum androgen levels and follicular development in low responders undergoing in vitro fertilization (IVF), although the number of studies is limited. The aim of the current study was to assess the association between basal serum androgen concentrations and the number of follicles ≥ 11 mm on the day of triggering final oocyte maturation in low responders undergoing IVF. This prospective study was performed from June 2020 to September 2024 in 96 low responders, categorized according to the Bologna criteria. Total testosterone, dehydroepiandrosterone sulfate (DHEAS), 17-OH progesterone (17-OH-P), Δ_4_-androstenedione (Δ4-A), and sex hormone-binding globulin (SHBG) were measured on the day of initiation of ovarian stimulation. No association was found between basal serum testosterone (coef.: −0.002, *p* = 0.98), DHEAS (coef.: −0.096, *p* = 0.35), 17-OH-P (coef.: +0.086, *p* = 0.40), Δ_4_-A (coef.: −0.028, *p* = 0.79), and SHBG (coef.: +0.160, *p* = 0.12) concentrations and the number of follicles ≥ 11 mm on the day of triggering final oocyte maturation. The results of the current study challenge the usefulness of basal serum androgen measurements prior to ovarian stimulation in low responders as predictors of ovarian response.

## 1. Introduction

Retrieval of multiple oocytes is a key factor for optimizing success rates in assisted reproductive technologies (ARTs) [1,2,3]. This is mainly achieved by the exogenous administration of gonadotropins. Unfortunately, in a significant proportion of women undergoing ovarian stimulation for in vitro fertilization (IVF), the number of oocytes retrieved is low, which, in turn, leads to a suboptimal probability of pregnancy [4].

Numerous strategies have been proposed for the management of this particularly challenging group of patients, known as low responders, aiming to increase the number of recruitable antral follicles, as well as their response to gonadotropin administration [5]. Designing interventions that could achieve these goals, however, necessitates an understanding of the mechanisms that regulate follicular recruitment and growth.

Androgens play a crucial role in folliculogenesis through their interactions with androgen receptors (ARs), which are expressed on the ovarian granulosa cells of pre-antral and antral follicles [6]. In the early stages of follicular development, androgens stimulate primordial follicle activation, granulosa cell proliferation, and follicular recruitment by stimulating insulin-like growth factor 1 (IGF-1) signaling, which synergizes with follicle-stimulating hormone (FSH) to support follicular growth and reduce atresia [7,8]. Moreover, androgens contribute to the recruitment and growth of antral follicles by enhancing FSH receptor expression in the granulosa cells of pre-antral and antral follicles and improving follicular sensitivity to FSH [9,10,11].

However, androgens decline with age [12], reflecting the diminishing ability of the aging ovary to respond to gonadotrophin stimulation during IVF. It has been demonstrated that low basal serum androgen concentrations are a significant risk factor for poor oocyte yield and a decreased probability of pregnancy [13,14]. Moreover, it has been suggested that low responders more frequently show hypoandrogenemia compared to normal responders undergoing ovarian stimulation for IVF [15,16].

Considering the above, it has also been proposed that the decreased androgen concentrations observed with advancing female age [12,17] contribute to the frequent occurrence of a low ovarian response [18]. In fact, high androgen concentrations in the ovarian microenvironment have been shown to promote early follicular development and granulosa cell proliferation [9,19]. A limited number of studies have evaluated the association between basal serum androgen concentrations and follicular development in low responders treated with exogenous gonadotropins for IVF, with conflicting results [20,21,22].

The aim of the current study was to assess the association between basal serum androgen concentration and the number of follicles on the day of triggering final oocyte maturation in low responders undergoing IVF. 

## 2. Results

### 2.1. Patient Population and Cycle Characteristics

**Figure 1:**
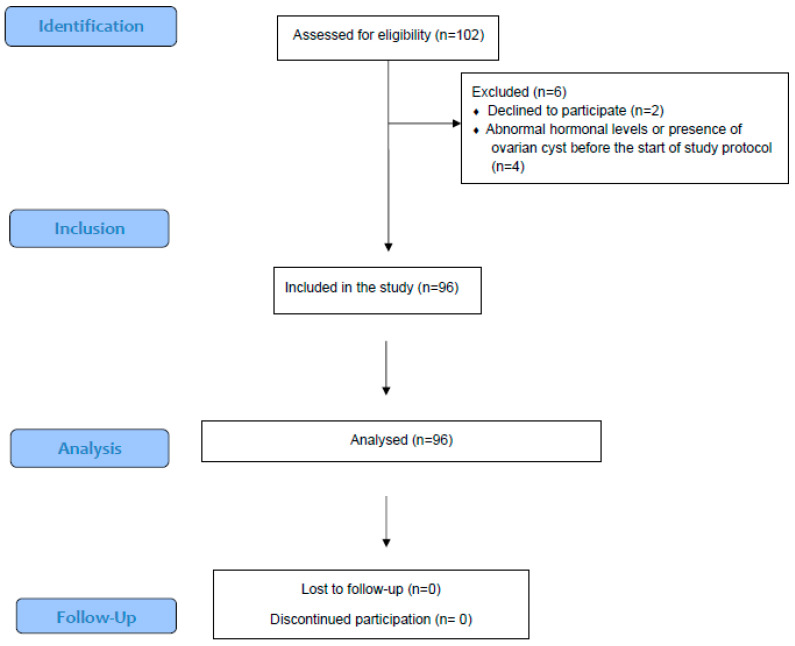
Flow diagram.

Ninety-six low responders fulfilling the Bologna criteria were recruited in this study (Figure 1). The demographic characteristics, basal hormone profile, and cycle characteristics are presented in Table 1.

Seven patients did not reach oocyte retrieval due to having no response to ovarian stimulation (7.3% 95% CI: 3.5–14.7% cancellation rate). In ten patients, no oocytes were retrieved (10.4%, 95% CI: 5.6–18.5%), while in six patients no fertilization was achieved (5.2%, 95% CI: 2.1–12.1%), and in twenty-three patients, embryo development failed (25.0%, 95% CI: 17.2–34.8%) (Table 1).

### 2.2. Primary Outcome Measure

**Figure 2:**
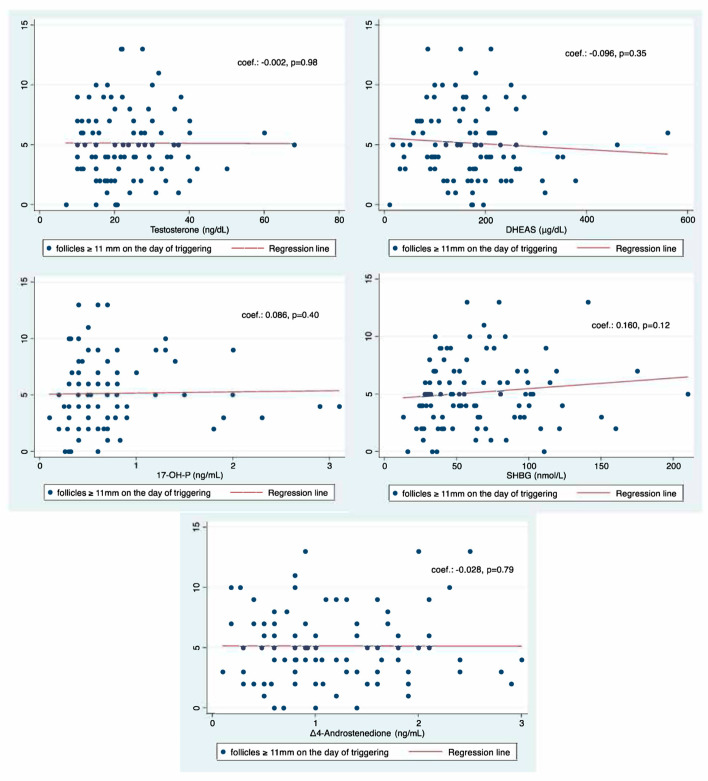
Linear regression between serum androgen (testosterone, DHEAS, Δ4-A, and 17-OH-P) and SHBG concentrations and the number of follicles ≥ 11 mm on the day of triggering final oocyte maturation.

No association was found between basal serum testosterone (coef.: −0.002, *p* = 0.98), DHEAS (coef.: −0.096, *p* = 0.35), 17-OH-P (coef.: +0.086, *p* = 0.40), Δ_4_-androstenedione (coef.: −0.028, *p* = 0.79), and SHBG (coef.: +0.160, *p* = 0.12) concentrations and the number of follicles ≥ 11 mm on the day of triggering final oocyte maturation (Figure 2). Moreover, no association was found in the multivariable regression analysis between basal androgen concentrations and the number of follicles ≥ 11 mm on the day of triggering final oocyte maturation, controlling for age, BMI, AFC, and AMH (Table 2).

### 2.3. Secondary Outcome Measures

No association was observed between basal serum testosterone (coef.: +0.024, *p* = 0.82), DHEAS (coef.: +0.017, *p* = 0.87), 17-OH-P (coef.: +0.191, *p* = 0.06), Δ_4_-androstenedione (coef.: +0.049, *p* = 0.64), and SHBG (coef.: +0.151, *p* = 0.14) concentrations and the number of follicles ≥ 17 mm on the day of triggering final oocyte maturation.

Furthermore, no association was observed between basal serum androgen concentrations and the number of COCs retrieved, the number of MII oocytes, and the number of 2pn oocytes (Table 3) by performing Spearman’s correlation analysis.

### 2.4. ROC Analyses

Basal serum androgen concentrations were not significant predictors of reaching oocyte retrieval (AUC 0.65, 95% CI: 0.49–0.81 for testosterone; AUC 0.60, 95% CI: 0.43–0.77 for DHEAS; AUC 0.76, 95% CI: 0.57–0.95 for 17-OH-P; AUC 0.58, 95% CI: 0.42–0.75 for Δ_4_-androstenedione; and AUC 0.66, 95% CI: 0.40–0.91 for SHBG).

**Figure 3:**
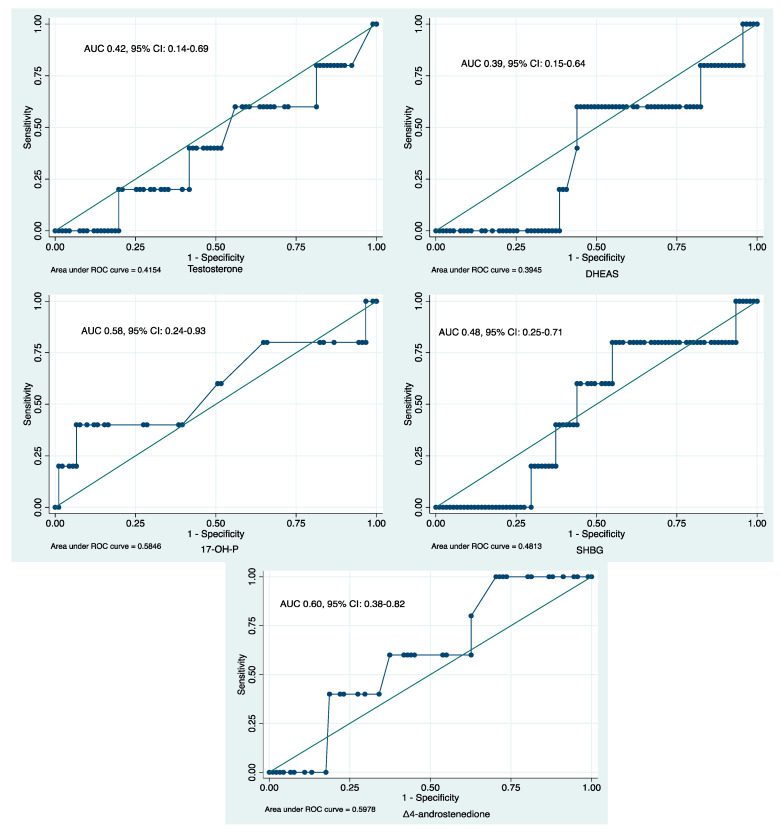
ROC curve analysis evaluating the discriminatory value of serum androgen concentrations for the achievement of a live birth.

Similarly, basal serum androgen concentrations were not significant predictors for the retrieval of ≤3 oocytes (AUC 0.55, 95% CI: 0.43–0.68 for testosterone; AUC 0.48, 95% CI: 0.35–0.60 for DHEAS; AUC 0.52, 95% CI: 0.40–0.63 for 17-OH-P; AUC 0.45, 95% CI: 0.33–0.58 for Δ_4_-androstenedione; and AUC 0.57, 95% CI: 0.46–0.69 for SHBG) or a live birth (AUC 0.42, 95% CI: 0.14–0.69 for testosterone; AUC 0.39, 95% CI: 0.15–0.64 for DHEAS; AUC 0.60, 95% CI: 0.38–0.82 for Δ_4_-androstenedione; AUC 0.58, 95% CI: 0.24–0.93 for 17-OH-P; and AUC 0.48, 95% CI: 0.25–0.71 for SHBG) (Figure 3).

## 3. Discussion

This prospective cohort study showed that basal serum androgen concentrations were not associated with follicular development on the day of triggering final oocyte maturation in low responders, as defined by the Bologna criteria, undergoing ovarian stimulation for IVF. Moreover, basal serum androgen concentrations were not able to discriminate between patients who had ≤3 oocytes retrieved or not and those who achieved a live birth or not.

To the best of our knowledge, this is the first prospective study including only low responders fulfilling the Bologna criteria and evaluating the association between basal serum androgen concentrations and the number of follicles on the day of triggering final oocyte maturation. However, the current study was not powered to assess the association of basal serum androgen concentrations with pregnancy outcomes.

Three previous studies have evaluated the association between basal serum androgen concentrations and follicular development in low responders undergoing ovarian stimulation for IVF, with conflicting results [20,21,22]. Two of these studies, retrospective in their design, showed that serum testosterone levels were positively associated with the number of follicles with a mean diameter >14 mm developed on the day of triggering final oocyte maturation, alongside pregnancy achievement [20,21]. On the other hand, the remaining prospective study showed that testosterone and DHEAS levels were not predictive of the implantation rate [22].

It should be noted that the above studies used different definitions of low ovarian response, which unfortunately leads to challenges in data synthesis and interpretation. To address this problem, the European Society of Human Reproduction and Embryology introduced the Bologna criteria [23], aiming to promote research on a more homogeneous population. Although the Bologna criteria have also been criticized in terms of the heterogeneity of the subpopulations they include [24], they still represent a significant step in the right direction [25].

Furthermore, relevant studies have focused only on the association of basal serum testosterone and DHEAS concentrations with the number of follicles on the day of triggering final oocyte maturation, while the concentrations of other androgens, such as 17-OH-P, Δ4-A, and SHBG have so far not been assessed. In the current study, a complete androgen panel was studied, allowing for a more holistic evaluation of the predictive value of androgens during gonadotrophin administration for follicular development.

**Figure 4:**
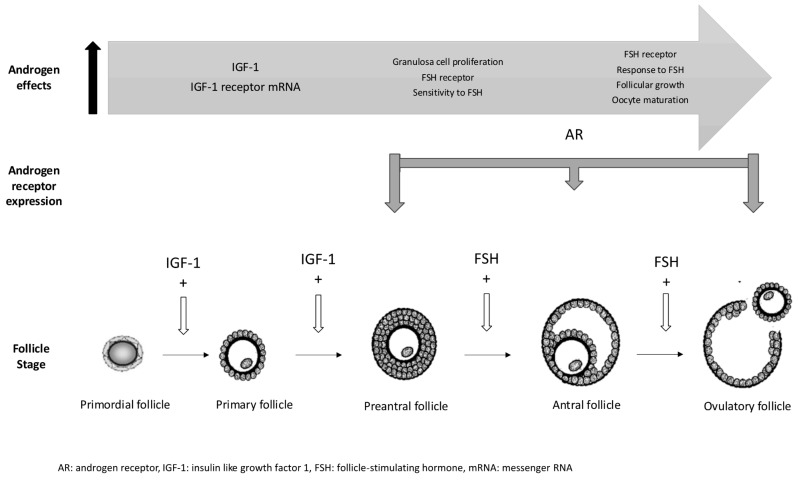
Androgen effects on follicular growth and development.

The decline in androgen bioavailability with age significantly impacts ovarian function, contributing to follicular atresia and reduced fertility [8,26]. The loss of androgen action leads to the decreased expression of receptors such as FSH and the insulin growth factor-1 (IGF-1) receptor, along with increased apoptosis in granulosa cells. At the molecular level, AR signaling impacts multiple aspects of folliculogenesis and its activation plays a critical role in regulating follicular growth. In AR knockout models, where androgen signaling is disrupted, there is an increase in follicular atresia, as shown by the presence of pyknotic granulosa cells, indicating the loss of follicular survival signals [26]. Moreover, androgens act by enhancing the responsiveness of the FSH receptor, a key receptor involved in follicular growth and maturation. The interplay between androgen and FSH receptor expression suggests that androgens not only support early folliculogenesis but also prime follicles for later stages of development [7,27]. The fact that AR expression in pre-antral follicles precedes FSH receptor expression supports the hypothesis that androgens potentially influence primordial follicle activation and survival. In addition, androgens influence the expression of growth factors such as IGF-1 and regulate the apoptotic pathways by modulating pro-apoptotic and anti-apoptotic genes, such as the expression of the anti-apoptotic microRNA (miR) miR-125b [26]. Therefore, this complicated molecular cross-talk between androgens, FSHR, and IGF-1 highlights the essential contribution of androgens to follicular growth, development, and maturation [28]. These molecular mechanisms between androgens, the FSH receptor, and IGF-1 highlight the essential contribution of androgens to follicular growth and maturation and their enhancement of ovarian responsiveness by improving reproductive outcomes in assisted reproductive technologies (Figure 4).

However, the results of the current study challenge the usefulness of basal serum androgen evaluation at the initiation of gonadotrophin administration in low responders as a predictor of multifollicular development. For this reason, the rationale of administering androgens during ovarian stimulation [29,30] based on the assessment of basal serum androgens should be revisited. Whether androgen administration might increase the probability of live birth independently of serum androgen levels upon the initiation of ovarian stimulation needs to be assessed in future prospective studies.

In conclusion, the current prospective cohort study did not show any association between basal serum androgen concentrations and follicular development on the day of triggering final oocyte maturation in low responders, categorized as such by the Bologna criteria, undergoing ovarian stimulation for IVF. Understanding the mechanisms of how androgens contribute to follicular growth provides valuable insights into tailoring treatment protocols to optimize the reproductive outcomes of poor responders. Future research should focus on improving androgen modulation strategies to enhance follicular growth and improve the probability of pregnancy in this challenging patient population.

## 4. Materials and Methods

### 4.1. Study Population

Low responders fulfilling at least two of the following three criteria (Bologna, 2010) [23] were included in this prospective cohort study: (i) advanced maternal age (≥40 years) or any other risk factor for poor ovarian response, (ii) a previous poor ovarian response (≤3 oocytes with a conventional stimulation protocol), and (iii) an abnormal ovarian reserve test, i.e., antral follicle count (AFC) < 5–7 follicles or anti-Mullerian hormone (AMH) < 0.5–1.1 ng/mL]. The exclusion criteria were endometriosis stage III–IV [31], a history of previous ovarian surgery, and endocrine or metabolic disorders.

This study was conducted in the Unit for Human Reproduction, in the 1st Department of Obstetrics and Gynaecology at the Aristotle University of Thessaloniki, from June 2020 to September 2024. This study was approved by the Ethics Committee Review Board of Papageorgiou General Hospital. Written consent was obtained from all the patients. Patients could participate in this study only once.

### 4.2. Hormonal Measurements and Ovarian Stimulation

Total testosterone, dehydroepiandrosterone sulfate (DHEAS), Δ_4_-androstenedione (Δ4-A) 17-OH progesterone (17-OH-P), and sex hormone-binding globulin (SHBG) were measured in the morning on the day of initiation of ovarian stimulation for each patient. Testosterone, DHEAS, and SHBG levels were measured by means of the Atellica^®^ IM Analyzer (Siemens Healthineers, Erlangen, Germany), whereas the 7-OH-P and Δ4-A levels were measured by means of the Active^®^ RIA (Beckman Coulter, Brea, CA, USA). The intra-assay and inter-assay coefficients of variation were <10% and <20% for testosterone, <10% and <13% for DHEAS, <7% and <20% for SHBG, <10.5% and <12.8% for 17-OH-P, and <7.5% and <11.3% for Δ4-A, respectively.

On day 2 of the menstrual cycle (stimulation day 1), patients were administered gonadotrophins at a fixed daily dose of 300 IU [32]. These included follitropin alfa (Gonal-F^®^; Merck Serono Europe Ltd., London, UK or Bemfola^®^; Gedeon Richter Plc, Budapest, Hungary), follitropin beta (Puregon^®^; NV Organon, Oss, The Netherlands), and menotropin (Menopur^®^; FERRING Pharmaceuticals S.A., St-Prex, Switzerland).

Starting on day 5 of stimulation, patients underwent monitoring with two-dimensional transvaginal ultrasound (2D TVS) and assessment of estradiol and luteinizing hormone (LH) every 2–3 days as required. The gonadotropin-releasing hormone (GnRH) antagonist ganirelix (Orgalutran^®^; NV Organon, Oss, The Netherlands) or cetrorelix (Cetrotide^®^; Merck Serono Europe Ltd., London, UK) was used to inhibit a premature LH surge, according to a fixed 5-day protocol. The “5-day protocol” referred to the initiation of GnRH antagonist on day 5 of ovarian stimulation with gonadotrophins, introduced in a fixed manner (regardless of follicle size) at a dose of 0.25 mg/0.5mL of cetrorelix or ganirelix, aiming to inhibit a premature LH surge. Follicular development was assessed by measuring the mean diameter of each follicle ≥11 mm by two-dimensional transvaginal ultrasound evaluation at each visit.

### 4.3. ART Procedure

Final oocyte maturation was triggered using 250 μg of recombinant human chorionic gonadotrophin (hCG) (Ovitrelle^®^, Merck Serono Europe Ltd., London, UK) as soon as at least three follicles ≥ 17 mm in diameter were present, or, if this was not possible, when one or two leading follicles were present that were between 17 mm and 24 mm in size, while optimizing the size of the remaining follicles with diameters between 11 mm and 17 mm.

Oocyte retrieval was performed transvaginally 36 h following the triggering of final oocyte maturation by puncturing all follicles ≥ 11 mm in size. Follicular flushing was not performed. Fertilization was performed either with conventional IVF or intracytoplasmic sperm injection (ICSI).

### 4.4. Outcome Measures

The primary outcome measure was the association between testosterone, DHEAS, Δ4-A, 17-OH-P, and SHBG concentration with the number of follicles ≥ 11 mm in diameter on the day of triggering final oocyte maturation. The secondary outcome measures included the association between androgen concentration and the number of follicles ≥ 17 mm on the day of triggering final oocyte maturation, the number of cumulus–oocyte complexes (COCs) retrieved, metaphase II (MII) oocytes, 2-pronuclei (2pn) oocytes, and the association between androgen concentration and the probability of reaching oocyte retrieval, the retrieval of ≤3 oocytes [23], and the achievement of a live birth.

### 4.5. Statistical Analysis

Variables were tested for normality with the Shapiro–Wilk test. Spearman correlation coefficient [coef. with 95% confidence interval (CI)], linear regression, and generalized linear model (GLM) analyses were used to investigate the association between androgen concentrations and the number of follicles on the day of triggering final oocyte maturation. Dichotomous variables were expressed as proportions (95% CI), whereas continuous variables were expressed as mean or median (95% CI), depending on the normality or not of the distribution. Statistical analyses were performed using STATA v14.0 (StataCorp., 2015, Stata Statistical Software: Release 14, College Station, TX, USA, StataCorp LP). Receiver operating characteristic (ROC) curve analyses were performed, aiming to evaluate the discriminatory value of serum androgen concentrations and binary outcomes, such as reaching oocyte retrieval, the retrieval of ≤3 oocytes, or the achievement of a live birth. A *p*-value < 0.05 was considered statistically significant.

### 4.6. Sample Size

Sample size estimation showed that 84 patients needed to be enrolled in this study in order to detect a correlation of at least 0.30, considering that values less than 0.30 indicated a weak correlation [33,34], using a two-sided hypothesis test with 80% power and a significance level of 0.05 [35]. To account for a potential 15% dropout rate, 96 patients were recruited in the current study.

## Figures and Tables

**Table 1:** Demographic and cycle characteristics.

Patient Characteristics (n = 96)	Median	95% CI
Age (years)	42.0	41.5–42.9
BMI (kg/m^2^)	24.1	22.5–25.7
AFC	6.0	5.0–7.0
AMH (ng/mL)	0.8	0.6–1.0
Serum androgen concentration		
Testosterone (ng/dL)	20.4	18.3–24.0
DHEAS (μg/dL)	170.1	148.0–180.0
17-OH-P (ng/mL)	0.6	0.5–0.6
Δ4-A (ng/mL)	1.0	0.9–1.2
SHBG (nmol/L)	55.7	47.0–64.6
Ovarian stimulation characteristics		
Duration of stimulation (days)	10.0	9.0–10.0
Total dose of FSH (IU)	3000	2700–3000
Day of triggering final oocyte maturation		
Number of follicles ≥ 11 mm	5.0	4.0–5.1
Number of follicles ≥ 17 mm	2.0	2.0–3.0
Estradiol (pg/mL)	1154	921–1285
IVF outcome		
COCs retrieved	3.0	2.0–4.0
MII oocytes	3.0	2.0–3.0
2pn oocytes	2.0	2.0–2.0
	% (n)	95% CI
IVF ICSI	20.8 (16) 79.2 (61)	13.0–31.5 68.5–87.0
Patients reaching ET	52.1 (50)	42.0–62.0
Positive hCG test	9.4 (9)	4.9–17.2
Ongoing pregnancy	5.2 (5)	2.1–12.1
Live birth	5.2 (5)	2.1–12.1

BMI: body mass index; FSH: follicle-stimulating hormone; AFC: antral follicle count; AMH: anti-Mullerian hormone; DHEAS: dehydroepiandrosterone sulfate; Δ4-A: Δ_4_-androstenedione; 17-OH-P: 17-OH progesterone; SHBG: sex hormone-binding globulin; IVF: in vitro fertilization; ICSI: intracytoplasmic sperm injection; COCs: cumulus–oocyte complexes; MII: metaphase II oocyte; 2pn: 2-pronuclei oocyte; hCG: human chorionic gonadotrophin and ET: embryo transfer.

**Table 2:** Multivariable analysis with the number of follicles ≥ 11 mm on the day of triggering final oocyte maturation as the dependent variable and basal serum androgen concentration, controlling for age, BMI, AFC, and AMH, as the independent variable.

Androgen and SHBG	Coef.	*p*-Value	95% Confidence Interval
Testosterone	+0.003	0.60	−0.007 to +0.013
DHEAS	−0.001	0.30	−0.002 to +0.001
17-OH-P	+0.015	0.89	−0.197 to +0.227
Δ4-A	−0.017	0.91	−0.300 to +0.267
SHBG	+0.002	0.24	−0.001 to +0.005

Coef.: coefficient; DHEAS: dehydroepiandrosterone sulphate; SHBG: sex hormone-binding globulin; Δ4-A: Δ_4_-androstenedione; 17-OH-P: 17-OH progesterone; AFC: antral follicle count; AMH: anti-Mullerian hormone; and BMI: body mass index.

**Table 3:** Spearman’s correlation analysis for the association between basal androgen concentrations and the number of COCs retrieved, as well as MII and 2pn oocytes.

	COCs	MII	2pn
	Coefficient *p*-Value		
Testosterone	−0.118 0.30	−0.176 0.12	−0.129 0.26
DHEAS	−0.041 0.72	−0.039 0.73	+0.060 0.60
17-OH-P	−0.008 0.95	−0.039 0.73	−0.103 0.37
Δ4-A	−0.036 0.75	−0.070 0.54	−0.045 0.69
SHBG	+0.143 0.21	+0.091 0.42	+0.010 0.93

DHEAS: dehydroepiandrosterone sulphate; SHBG: sex hormone-binding globulin; Δ4-A: Δ_4_-androstenedione; 17-OH-P: 17-OH progesterone; COCs: cumulus–oocyte complexes; MII: metaphase II oocyte; and 2pn: 2-pronuclei oocyte.

## Data Availability

Data available upon request.
